# Risks of testosterone therapy in elderly men

**DOI:** 10.12688/f1000research.3-11.v1

**Published:** 2014-01-15

**Authors:** Ranjith Ramasamy, James M. Dupree, Jason R. Kovac, Larry I. Lipshultz

**Affiliations:** 1 Department of Urology, Baylor College of Medicine, Houston, TX 77030, USA

## Abstract

Testosterone supplementation therapy (TST) is a widely used treatment for men with late onset hypogonadism. The benefits seen with TST, such as improved libido and energy level, beneficial effects on bone density have been well documented. Although hypogonadism remains an independent risk factor for mortality, recent studies have examined the association between testosterone therapy and cardiovascular risk.

## Correspondence

Vigen *et al.* ^1^ examined the association between testosterone supplementation and cardiovascular morbidity in men older than 60 years. They performed a retrospective national cohort study of men with low testosterone levels (<300 ng/dL) who underwent coronary angiography in the Veterans Affairs (VA) system between 2005 and 2011. The absolute rate of atherosclerotic events (myocardial infarction, stroke and mortality) was 19.9% in men who did not receive testosterone vs 25.7% in the men who were treated with testosterone. Men on testosterone supplementation were reported to have higher risk of adverse events than men not on testosterone, despite being younger and having less comorbidity.

One of the most important messages to glean from the study is that hypogonadism could be an adverse prognostic factor for cardiovascular and cerebrovascular morbidity and mortality. This message is also found in other studies about hypogonadism, including a large study on male veterans that showed that hypogonadism could be an important risk factor for increased mortality ^2^. Further, in men with hypertension ^3^, low testosterone levels were shown to be associated with increased risk of major cardiovascular adverse events.

In the Vigen *et al.* study men who received testosterone had lower pre-therapy testosterone levels, suggesting that they were even more hypogonadal than men who did not start testosterone therapy.In addition, it is unclear how much testosterone the men in the treatment arm actually received. Based on prescription refills, most men were on testosterone therapy for less than one year, and their mean post-treatment testosterone level was 332 ng/dL. With serum testosterone less than 300 ng/dL defined as biochemical hypogonadism by the Endocrine Society ^4^, we are concerned that a significant proportion of men could have remained hypogonadal, in spite of testosterone treatment.

Additionally, the reasons for starting testosterone therapy cannot be determined from this retrospective analysis. Because of the uncertain reasons for starting therapy in some hypogonadal men and not in others, and because of the variability in the amount of total testosterone that the patients actually received, there may be confounding factors that could also explain the higher risk of adverse events in men treated with testosterone. It is unclear whether the minimal exposure to testosterone in this elderly population (as evidenced by the post-treatment levels and duration of treatment) was responsible for such a dramatic difference in mortality and morbidity.

The association between testosterone therapy and mortality has remained controversial with studies demonstrating conflicting results ^5, 6^. Until larger randomized studies demonstrate clear causation, physicians prescribing testosterone therapy to elderly men with co-morbidities should use it prudently with close follow-up.
