# Supplementary material for: Risks of testosterone therapy in elderly men
Source: F1000Res. 2014 Jan 15;3:11. [Version 1] doi: 10.12688/f1000research.3-11.v1 (PMC3901445; doi:10.12688/f1000research.3-11.v1)
Supplement: Peer review history

# Peer review: Referee response for version 1

**Published:** 2014-01-23

**Authors:** Matthew S. Wosnitzer

**Affiliations:** 1 Center for Male Reproductive Medicine and Surgery, Department of Urology and Institute for Reproductive Medicine, Weill Cornell Medical College of Cornell University, New York, NY, USA

This correspondence from the Baylor group accurately summarizes many of the questions surrounding the Vigen *et al.* article which describes an increased cardiovascular risk profile with testosterone replacement in a highly select group of VA patients. The majority of prior studies have not detected significant increases in cardiovascular risks, except for the randomized TOM trial ( Basaria *et al. *2010 NEJM), which had many of its own issues with *"differences detected between the two trial groups possibly...due to chance alone."*

One key criticism of the Vigen *et al.* study, described in this correspondence, is that hypogonadism alone may portend adverse cardiovascular and cerebrovascular outcomes and that the T treatment group in this study had lower pre-treatment T values. This issue is most recently highlighted in the JCEM article by Yeap *et al* *.* from Australia in 2013 describing older Australian men with reduced mortality when testosterone was in mid-range normal levels.

Other issues with the Vigen *et al.* study include the heterogeneous preparations of testosterone, and varied/unknown compliance with regimens. Only 60% of patients had follow-up testosterone labs after starting therapy which is particularly important since T response differs from regimen to regimen and patient to patient. Additionally for those who had testosterone checked, were they done appropriately in the morning or at other times? Additionally, known mean post-treatment testosterone levels were barely in eugonadal range, further highlighting possible compliance issues. Certainly, additional randomized controlled trials are required to discern whether the findings in this population are generalizable to any other population.

I have read this submission. I believe that I have an appropriate level of expertise to confirm that it is of an acceptable scientific standard.

# Peer review: Referee response for version 1

**Published:** 2014-01-22

**Authors:** Boback Berookhim

**Affiliations:** 1 Memorial Sloan Kettering Cancer Center, New York, NY, USA

Dr. Ramasamy and colleagues present a well-researched brief summary on a topic which has received great attention among physicians treating low testosterone. The authors provide a nice summary of a newsworthy publication by Vigen *et al.* in JAMA in late 2013. The concerns presented with the methodology of this article, primarily regarding the efficacy of likely sub-therapeutic testosterone replacement therapy in an elderly veteran population, are relevant and worthy of further study. Given the conflicting studies within the literature, further research is required to clearly delineate the cardiovascular risks (or lack thereof) in elderly men receiving testosterone replacement therapy.

I have read this submission. I believe that I have an appropriate level of expertise to confirm that it is of an acceptable scientific standard.
